# Retailers' knowledge of tobacco harm reduction following the introduction of a new brand of smokeless tobacco

**DOI:** 10.1186/1477-7517-7-18

**Published:** 2010-07-29

**Authors:** Karyn K Heavner, Zale Rosenberg, Francis Tenorio, Carl V Phillips

**Affiliations:** 1 School of Public Health, University of Alberta, Edmonton, Alberta, T6G 2L9, Canada; 2 TobaccoHarmReduction.org, Saint Paul, MN, 55104, USA

## Abstract

**Background:**

Tobacco retailers are potential public health partners for tobacco harm reduction (THR). THR is the substitution of highly reduced-risk nicotine products, such as smokeless tobacco (ST) or pharmaceutical nicotine, for cigarettes. The introduction of a Swedish-style ST product, du Maurier snus (dMS) (Imperial Tobacco Canada Limited), which was marketed as a THR product, provided a unique opportunity to assess retailers' knowledge. This study examined retailers' knowledge of THR and compliance with recommendations regarding tobacco sales to young adults.

**Methods:**

Male researchers, who may have looked younger than 18 years old, visited 60 stores in Edmonton that sold dMS. The researchers asked the retailers questions about dMS and its health risks relative to those from other tobacco products. They also attempted to purchase dMS to ascertain whether retailers would ask for identification to verify that they were at least 18 years old.

**Results:**

Overall, the retailers were only moderately knowledgeable about THR and the differences between dMS and other tobacco products. About half of the retailers correctly indicated that snus is safer than cigarettes; half of whom knew it is safer because it is smoke-free. Fifty percent incorrectly believed that snus causes oral cancer. Less than fifty percent indicated that dMS differs from chewing tobacco because it is in pouches and is used without spitting or chewing (making it more promising for THR). Most (90%) of the retailers asked the researchers for identification when selling dMS.

**Conclusion:**

Tobacco retailers are potentially important sources of information about THR, particularly since there are restrictions on the promotion of all tobacco products (regardless of the actual health risks) in Canada. This study found that many retailers in Edmonton do not know the relative health risks of different tobacco products and are therefore unable to pass on accurate information to smokers.

## Background

The availability of accurate tobacco harm reduction (THR) information at locations where smokers purchase cigarettes is largely unknown but has great public health importance. THR, the substitution of lower risk sources of nicotine for smoking, is a promising intervention for smokers who will not quit nicotine or tobacco entirely [1-4]http://tobaccoharmreduction.org. Almost all the risk from smoking comes from inhaling chemicals produced during the combustion of organic matter, not from nicotine or the tobacco plant itself. It is because of this that non-combustion sources of nicotine, such as smokeless tobacco (ST) and pharmaceutical nicotine products cause roughly 1/100^th ^the risk of life-threatening disease from cigarettes [5]. Electronic cigarettes probably have approximately the same mortality risks (because users do not inhale combustion products) but have not been studied as extensively. The ability of smokers to make an informed, autonomous choice about whether to keep smoking, switch to less harmful nicotine products, or stop using nicotine entirely, should be based on accurate information about the products, including information about the relative health risks of the different products. Documented misperceptions about THR include the beliefs that: ST poses the same or greater health risks as smoking; ST has been shown to cause a measurable risk of oral cancer (typically colloquially phrased as "ST causes oral cancer"); and the smoke itself is not the source of most of the health risks from smoking [6-12]. Accurate knowledge about ST products is especially important for retailers who interact with customers purchasing tobacco products, and may prevent or contribute to the propagation of disinformation. This is particularly true in Canada because of the near prohibition on the manufacturers' ability to communicate health information to their customers other than in-person at the point of sale, and restrictions on the right to free speech that criminalize even private provision of accurate information about tobacco products.

The introduction of a new Swedish-style ST product, du Maurier snus (dMS), by Imperial Tobacco Canada Limited (ITC) (a subsidiary of British American Tobacco) in 2007 provided a unique opportunity to assess retailers' knowledge of THR and the sale of ST to young adults. Snus is the Swedish term for pasteurized moist snuff that is usually sold in small sachets that users place between their upper lip and gum and du Maurier is the brand name of one of ITC's premium cigarettes. Other ST products (mainly US Smokeless Tobacco Company's moist snuff products) were widely available in Edmonton prior to the launch of this product [13,14]. The marketing strategy for dMS differs from that for other ST products because ITC is marketing it explicitly to their and other companies' cigarette customers as a harm reduction product. Around the time of the rollout retailers were educated about the product category and provided with a brochure, entitled "What is SNUS" to distribute to adult customers, particularly those purchasing tobacco products. They also received oral briefings by sales representatives of ITC and some of them attended an educational/social event at the time of the product rollout. The dMS product displays were quite prominent at the time of the rollout and data collection [13] before a provincial legal change mandated that no tobacco products could be visible to consumers. The display consists of a small refrigerator, usually located behind or beside the cashier.

Our study examined retailers' knowledge of the comparative risks of different tobacco products and other health information about ST; information that they received in oral briefings and written materials about dMS. In addition, we took advantage of the study to also examine compliance with recommendations regarding the sale of tobacco to young adults. According to recommendations from Operation I.D., which provides materials about the sale of tobacco products to youth, retailers should ask individuals who *appear *to be under the age of 25 for identification before selling any tobacco product [15].

## Methods

A list of the 219 retail outlets in the Edmonton area where dMS was sold at the time of the study was obtained from ITC. Fifty-two outlets outside of the city of Edmonton were excluded to simplify the logistics of data collection so that the study could be completed in a timely manner. A random sample of 60 of the remaining 167 stores in the city of Edmonton was selected. Two male undergraduate students (two of the authors (FT and ZR)), hereafter referred to as researchers, aged 20 and 21 were trained to approach the retailers, ask questions about THR as part of a conversation about dMS, and attempt to purchase dMS. The dMS refrigerator was often near the cash register, allowing for a visual reference to the product. The researchers were greater than the legal age to purchase tobacco in Alberta but sufficiently young-looking that they should have triggered the "check identification if under 25" recommendation. No female students were included because males are much more likely to use ST (e.g., [9,16]), and thus appeared more natural. The researchers dressed in casual clothes (e.g., jeans and sweatshirts).

In each store, one researcher approached a cash register and asked the nearest employee a series of questions about the health risks of dMS and THR. The researcher then purchased one container of dMS, showing his Alberta driver's license if he was asked for identification. As it was crucial for the researchers to appear as normal customers rather than researchers, they did not follow the script exactly, but rather rehearsed following naturally flowing conversations and asking their questions at appropriate opportunities. The researchers completed a data collection form as soon as possible after leaving each retail outlet, as doing so inside the store might have affected the employee's interactions with the researcher. The script, data collection form and a de-identified version of the data are available at http://tobaccoharmreduction.org/research/retailer.htm

After data collection was completed, the responses to each question were categorized based on the correct answers. These categories are described in the discussion section to provide the necessary regulatory and ideological framework for the retailers' responses. SAS (version 9.1, SAS Institute, Cary, North Carolina) was used for the sample selection and data analysis.

Retailers' consent was not obtained for this study. Our goal was to observe the retailers' behavior during the course of their normal jobs, and asking for consent would have prevented this. Asking for consent would have necessitated limiting the study to an assessment of the retailers' responses to what they knew was, in effect, an exam, and would have prohibited any assessment of whether retailers' appropriately asked seemingly underage customers for identification. The retailers were later sent a letter and fact sheet describing the study. The study protocol was reviewed and approved by the Health Research Ethics Board at the University of Alberta.

## Results

**Figure 1:**
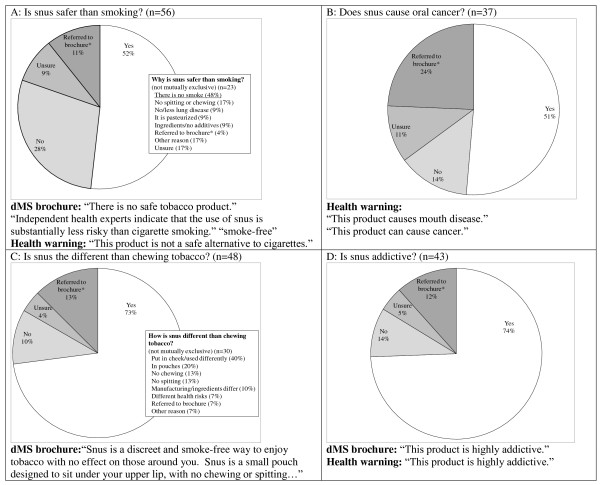
Relevant information in the dMS brochure and the four alternating federally mandated warnings that take up half of the front of the dMS packages.

Data collection was completed in February and March 2008. One researcher visited 39 stores, while the other visited 21 stores. All visits occurred during weekdays between 9 am and 5 pm. Most of the outlets were convenience stores. The researchers did not ask questions about dMS or THR in two stores where tobacco company representatives were present. Two retailers refused to answer any questions about the product and an additional four, including one who did not appear to speak English well, did not answer any questions but gave the researchers the dMS brochure. In one store there was a handwritten information sheet about snus on the dMS refrigerator. Retailers' answers to specific questions about dMS and THR are illustrated in Figure 1. Relevant information in the dMS brochure and the four alternating federally mandated warnings that take up half of the front of the dMS packages are also listed in Figure 1 to help frame the retailers' responses.

### Is snus safer than smoking?

Only about half of the retailers correctly stated that snus is safer than smoking. One retailer stated that it is 99% safer but the rest gave no indication of the magnitude of the risk difference. Only about half of the retailers who were aware that snus is safer attributed the risk reduction to the lack of smoke. One quarter of the retailers who did not believe that snus is safer indicated that it is not safer because it is a tobacco product. An additional 19% indicated that it is not safer because it causes oral cancer or mouth diseases.

### Does snus cause oral cancer?

Fifty percent of the retailers who were asked and answered questions about this topic told the researchers that snus causes oral cancer. Three retailers did indicate that smoking also causes oral cancer or that snus is less likely to cause oral cancer than smoking. Many of the retailers did not respond to this question or were not asked this question because it could not be raised as part of an inconspicuous conversation.

### Is snus different than chewing tobacco?

Most (73%) of the retailers indicated that snus is different than chewing tobacco and many correctly identified that the differences relate to the use of the product and not the health risks. Many of the retailers referred to other moist snuff products, which were available in most of the stores, as chewing tobacco.

### Is snus addictive?

Three-quarters of the retailers believed that snus is addictive, which is stated in the brochure and one of the warnings on the package ("This product is highly addictive.").

### Researchers' attempts to purchase snus

Snus was purchased in all but three of the stores. Two of the locations did not have any dMS in stock. A retailer who was speaking with a representative from a company that markets a competing ST product when the researcher entered the store claimed that the product was not available (even though the dMS was clearly visible to the researcher) and did not sell dMS to the researcher. A representative from ITC was in one store where snus was purchased.

Table 1 describes the researchers' experiences attempting to purchase dMS in the 58 stores where tobacco company representatives were not present. Most (90%) of the retailers asked the researchers for identification to verify their age. All of the retailers who did not ask for identification either answered the researchers' questions about snus or gave them the brochure. Five retailers who did not ask for identification sold dMS to the researchers. Thirty-nine percent of the retailers who asked for identification did so before answering questions about the product, 41% before the transaction and 20% during the transaction. In one store, the retailer initially questioned the validity of the researcher's identification but upon follow-up did sell dMS to him. The researchers were not given the snus brochure in approximately one-third of the stores (retailers in one-third of these stores said that they had run out of the brochures).

**Table 1:** Sale of snus to young adults who may appear to be <18 years old (n = 58)*

	n	%
Researcher purchased snus		
Yes	56	97%
No	2	3%
		
Retailer asked to see identification		
Yes	52	90%
No	6	10%
		
Researcher received snus brochure		
Yes, without requesting it	28	48%
Yes, but had to request it	11	19%
Brochures were placed so customers could take them	1	2%
No	18	31%

* Excludes the two stores where tobacco company representatives were present. Snus was purchased in one of these stores.

## Discussion

Retailers' misperceptions were consistent with the confusing and often inaccurate information about ST in the mandated health warnings, the dMS brochure, popular press and on the internet. Additional information may have come from public statements to the community by our research group, particularly a presentation by one of us (CVP) at an educational/social event organized by ITC prior to the product launch. In particular the specific estimate of 99% risk reduction and the fact that smoking is much more likely to cause oral cancer than snus use are common in our communications but not ITC's, and thus most likely trace specifically to us.

Retailers may have a rapport with cigarette customers who they see frequently and may respond differently to those individuals. However, it is possible that the researchers' experiences may be similar to those of young smokers who are interested in reduced harm nicotine products. Many of the retailers were hesitant to speak with the researchers or did not answer their questions. Retailers working alone were more likely to engage in a conversation with the researchers than if there were other employees or customers in the vicinity. Obviously, if retailers suspected that the researchers were "secret shoppers" (underage youths attempting to purchase tobacco to see if retailers asked them for identification), their interactions with the researchers might have been different than with other young adults. We had no clear indications that this was the case, but it was possible.

### Is snus safer than smoking?

Retailers' responses to this question were consistent with the potentially confusing information about dMS in the brochure, on the package, in the media at the time of the product launch [17], and misleading and incorrect information about ST online [18,19]. The claim that ST is not safe or is not safer than smoking is common, as evidenced by the dMS brochure and health warning on the dMS package. It is clearly confusing to consumers and it is likely that retailers are no more sophisticated, mistakenly confusing "not safe" with "not much safer than cigarettes."

The common assertion that ST products are not "safe" is counterproductive. The statement in the dMS brochure that there is "no safe tobacco product" and the similar health warning are literally true, but highly misleading given how small the risk from ST is compared to smoking (approximately 1/100^th ^the mortality risk [18]). It is not surprising that many retailers did not know that snus is *safer *than cigarettes. The brochure did not make an explicit link between the risk reduction and the lack of smoke. The attribution of the risk reduction to things other than the lack of smoke is consistent with previous research which found that smokers often attribute the health risks of cigarettes to things other than the smoke (such as additives, nicotine, or the other natural components of tobacco itself) [6-8].

### Does snus cause oral cancer?

Although the belief that ST causes oral cancer is a common misconception, experts agree that the epidemiology clearly shows that if there is any oral cancer risk from snus or other modern Western ST products, it is too small to measure [2,3,20,21]. The majority of cases of oral cancer in North America are likely attributable to a combination of smoking and alcohol consumption [22]. Two of the mandated warnings on the dMS package may have contributed to retailers' confusion about oral cancer. ST use does cause superficial irritations in many users but these lesions are different than those caused by smoking and very rarely become cancerous [20].

### Is snus different than chewing tobacco?

The main differences between snus and chewing tobacco in terms of usage are that: 1) dMS is in sachets instead of loose tobacco, making it less messy to use; 2) while placement is up to the individual, snus is typically placed between the upper lip and gum (made easier by the sachet that keeps the product from moving or disbursing), whereas chewing tobacco is typically held in the lower cheek area and loose snuff is usually used between the lower lip and gum; and 3) placement under the upper lip eliminates or minimizes the need to spit. In addition, it is heat-treated (pasteurized), which snus manufacturers sometimes claim reduces its health risks compared to other ST products, a claim that is plausible but not actually supported by the current evidence [23]. The evidence is not sufficient to distinguish between the low risks of moist snuff (including snus), chewing tobacco, and pharmaceutical nicotine products.

### Is snus addictive?

The retailers' beliefs about the snus being addictive are consistent with the brochure and one of the warnings on the package. It is true that snus, like all tobacco products, contains nicotine which is considered to be addictive. Thus, it seems reasonable that the retailers should have answered "yes," and this is reasonable shorthand for the accurate observation that many users of nicotine (from any source) become inveterate users. They would not be expected to offer nuances or know that "addiction" is not actually well-defined [24,25], that many definitions of addictive chemicals do not include nicotine [25] and that nicotine consumption may be beneficial for some people [25-27].

### The sale of snus to young adults who may appear to be minors

A common argument against THR is the claim that promoting it will increase the chance that ST products will be used by minors [28,29]. Most studies regarding the sale of tobacco products to minors focused on cigarettes [30-33], but there are some claims that retailers may be more likely to sell ST products to minors [30,34]. Although such claims seem to be of relatively minor importance (why worry so much about minors getting low-risk nicotine products given how many of them choose to and are able to smoke), it is still interesting to investigate.

## Conclusions

The promotion of low-risk nicotine products as an alternative to smoking may depend largely on information provided by retailers. This is the case because the environment is characterized by manufacturers having limited opportunities to communicate to customers, there is limited communication of accurate information from the scientific community and inaccurate and misleading information is often issued by anti-tobacco groups and governmental and non-governmental organizations. Our study suggests that despite efforts to educate retailers, they lacked some combination of the time, knowledge, or analytic sophistication to provide several of the key bits of information needed to explain the value of THR. While some retailers provided useful and accurate information, many did not. Lack of accurate information about THR is not surprising given the misinformation in the popular press[17], and on the internet [18,19]. It is somewhat disappointing, though not necessarily surprising, that retailers who either received directed education or could have been educated by other staff members on the point shared the popular misperceptions. The misleading or unclear warning statements on ST packages probably contributed to this, and the equivocal claims in the dMS brochure may have also contributed.

Regulatory changes occurred subsequent to the introduction of dMS (which we detail elsewhere [14]), including prohibiting the display of snus or informational brochures. It is unlikely that current customers would seek information like our researchers did, and if they did, the printed material would not be available. Thus, this study is probably more informative for markets where free speech at point-of-sale is still protected than it is about the current situation in Edmonton. The results from this study suggest that retailers in Edmonton may be contributing to public misperceptions about THR as much as they are reducing them. This suggests that other restrictions on free speech about THR -- advertising, package inserts, etc. - may be detrimental to the public health, since smokers' major remaining potential source of information is inadequate. The result is that even where actively providing accurate point-of-sale information is not criminalized and retailers are actively encouraged to provide the information, many smokers who might have quit by switching products will never learn about this potentially lifesaving option.

## List of abbreviations

ITC: Imperial Tobacco Canada; THR: Tobacco harm reduction; ST: Smokeless tobacco; dMS: du Maurier snus;

## Competing interests

The authors are interested in encouraging tobacco harm reduction (reducing the morbidity and mortality caused by tobacco use by encouraging smokers to switch to nonsmoked nicotine sources). As a result, they have an interest in designing research that explores smokers' access to accurate information about tobacco harm reduction products. In addition to this actual substantial interest, some people believe that conflict of interest stems from (and only from) funding rather than actual worldly goals. In response to this naive but common view that funding is more important than ethical beliefs and worldly goals, we report: Dr. Phillips and his research group (including Dr. Heavner, Mr. Rosenberg and Mr. Tenorio) are partially supported by an unrestricted (completely hands-off) grant to the University of Alberta from U.S. Smokeless Tobacco Company. The grantor is unaware of this manuscript, and thus had no scientific input or other influence on it. Dr. Heavner owns a small amount of stock in Johnson and Johnson. Dr. Phillips has consulted for U.S. Smokeless Tobacco Company in the context of product liability litigation and is a member of British American Tobacco's External Scientific Panel. Imperial Tobacco Canada Limited was not informed of this study until the debriefing letter and fact sheet were sent to the retailers, and had no scientific input or other influence on it.

## Authors' contributions

CVP and KH conceptualized the study and wrote the study protocol. FT and ZR collected the data that were analyzed by KH, FT and ZR. All authors contributed to writing the manuscript and reviewed it.
